# Structural analysis of *Clostridium botulinum* neurotoxin type D as a platform for the development of targeted secretion inhibitors

**DOI:** 10.1038/srep13397

**Published:** 2015-09-01

**Authors:** Geoffrey Masuyer, Jonathan R. Davies, Kevin Moore, John A. Chaddock, K. Ravi Acharya

**Affiliations:** 1 Department of Biology and Biochemistry, University of Bath, Claverton Down, Bath BA2 7AY, UK; 2 Ipsen Bioinnovation Limited, Units 4-10, The Quadrant, Barton Lane, Abingdon, Oxon OX14 3YS, UK; 3 Current address: Department of Biochemistry and Biophysics, Arrhenius Laboratories for Natural Sciences, Stockholm University, 10691 Stockholm, Sweden

## Abstract

The botulinum neurotoxin type D is one of seven highly potent toxins produced by *Clostridium botulinum* which inhibit neurotransmission at cholinergic nerve terminals. A functional fragment derived from the toxin, LHn, consisting of the catalytic and translocation domains, has been heralded as a platform for the development of targeted secretion inhibitors. These secretion inhibitors are aimed at retargeting the toxin towards a specific cell type to inhibit vesicular secretion. Here we report crystal structures of LHn from serotype D at 2.3 Å, and that of SXN101959 at 3.1 Å resolution. SXN101959, a derivative that combines LHn from serotype D with a fragment of the growth hormone releasing hormone, has previously revealed promising results in inhibiting growth hormone release in pituitary somatotrophs. These structures offer for the first time insights into the translocation domain interaction with the catalytic domain in serotype D. Furthermore, structural information from small-angle X-ray scattering of LHn/D is compared among serotypes A, B, and D. Taken together, these results demonstrate the robustness of the ‘LHn fold’ across serotypes and its use in engineering additional polypeptide components with added functionality. Our study demonstrates the suitability of botulinum neurotoxin, and serotype D in particular, as a basis for engineering novel secretion inhibitors.

Toxins from *Clostridium botulinum* species are the causative agent of the rare neuroparalytic illness botulism. Seven distinct serotypes (A–G) of botulinum neurotoxins (BoNTs) affect humans and other species to varying degrees. Once inside the neuronal cell, BoNTs block the release of neurotransmitters leading to paralysis^1^. Although they are highly toxic, various BoNTs are available commercially as therapeutic agents^2^.

BoNTs are synthesized as a single polypeptide chain (150 kDa), which is post-translationally cleaved into a di-chain molecule composed of light chain (LC, 50 kDa) and heavy chain (HC, 100 kDa). LC is the catalytic domain and a zinc-endopeptidase, while HC is further divided into two sub-domains of equal molecular mass called the translocation domain (Hn) and the membrane binding domain (Hc). On binding to the nerve terminals, BoNTs are endocytosed into a vesicle, where the acidic environment causes some conformational changes allowing LC to enter the cytosol^3^. Inhibition of neurotransmission takes place by proteolysis of one of the SNARE proteins^1^ that mediate cell secretion^4^.

Of the seven types of botulinum toxin, A, B, E and F are known to cause the disease in human while C and D have only been observed in animal cases. More particularly, D has been responsible for several recent outbreaks of botulism in cattle^5^. This has raised some interest in this serotype and understanding its precise mechanism of action. No cases of type D human botulism have ever been recorded. Coffield *et al*.^6^ investigated the impact of serotype D on human tissues and demonstrated its inability to block neuromuscular transmission when tested at a level 10 times higher than that of serotype A. This limited activity was recently confirmed *in vivo* by electrophysiological study on human muscles^7^ and may be linked to differences in the receptor binding domain^8^,^9^. BoNT/D however, acts similarly to the other botulinum neurotoxins by targeting one of the intracellular SNARE proteins. Synaptobrevin (or VAMP) was identified as the BoNT/D specific substrate^10^ and is cleaved at the Lys59-Leu60 position.

BoNT/A and /B are currently the only serotypes approved for medical uses. With the emergence of immuno-reactivity and resistance among patients^11^, other serotypes such as BoNT/D can provide a useful alternative. The successful retargeting of BoNT activity for therapeutic purposes, by association of the LHn fragment (catalytic and translocation domains) with various ligands has been described^12^. In particular, a targeted secretion inhibitor (TSI) combining a growth hormone releasing hormone (GHRH) receptor targeting domain with the LHn/D fragment, called qGHRH-LHn/D, was shown to specifically inhibit pituitary somatotroph growth hormone release^13^. This molecule demonstrated efficient intracellular activity on VAMP-3 in rat pituicytes and thus encouraging potential in the treatment of Acromegaly^14^. The latest LHn/D derivative, SXN101959, which combines the functional BoNT fragment with a GHRH ligand domain [qGHRH(1–40)], presented potent and reversible inhibitory action on the somatotropic axis and such features are well aligned with treating overproduction of growth hormone^15^. The arrangement of the functional domains within SXN101959 involves a novel orientation in which the GHRH ligand is located centrally between the LC and HC in the single chain polypeptide expressed in *E. coli* and with a protease cleavage site located between the GHRH ligand and the LC, such that following protease activation to generate the active di-chain TSI the GHRH ligand is at the amino-terminus of the HC domain. This arrangement, termed central presentation, was necessitated by the requirement of the GHRH ligand to have a free N-terminus to be able to bind and activate its receptor. It means, however, that the relative arrangement of the binding domain relative to the LC and Hn domains is different to that in BoNT and previously reported TSI proteins^16^. The resulting TSI protein is functional in respect to binding its receptor, internalising into the cytosol of target cells, cleaving its substrate SNARE protein and inhibition of growth hormone secretion^14^,^15^. However, the consequence of this novel domain arrangement upon the structure of the TSI protein is not known.

Structural and biochemical characterisation of the LHn fragments from serotypes A and B^17^,^18^ have provided a molecular basis for their functionality, representing an important step forward for the design of novel molecules based on these frameworks. In order to assess its applicability for further pharmaceutical development, the structure of LHn/D was analysed by X-ray crystallography (solid crystalline state) and small angle X-ray scattering (SAXS, in the solution state). The X-ray crystal structure showed key elements of BoNT/D including the flexibility of the translocation domain and the elaborate interaction with its catalytic partner. The SAXS analysis corroborates the relevance of the crystal structures and the stability of the LHn template in solution. The X-ray crystal structure of SXN101959 at 3.1 Å further confirmed the rigidity of LHn/D’s structural framework and its ability to accommodate novel arrangements of the domain subunits such as central presentation, although the GHRH ligand domain was not visible. This study should thus provide the structural basis for the development of BoNT/D-based Targeted Secretory Inhibitor (TSI) which could prove useful in the treatment of hypersecretory disorders.

## Results and Discussion

### Structure of LHn/D

**Figure 1:**
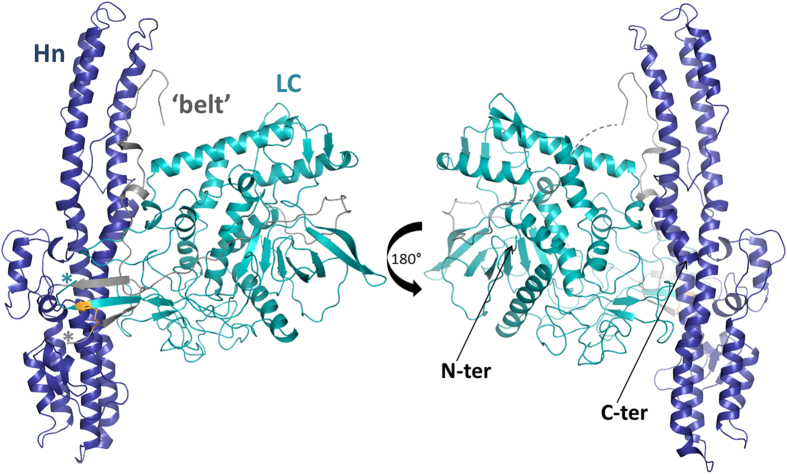
Crystal structure of LHn/D. Ribbon diagram representation of LHn/D structure, LC in cyan, belt region in grey and Hn in blue. C-terminus of LC and N-terminus of Hn are marked with a cyan and grey asterisk, respectively. The disordered region of the belt is presented as a grey-dashed line. The disulphide bond between LC and Hn is highlighted in orange.

This is a mutant endo-negative form of LHn/D in which the catalytic domain is inactivated by a two amino acid substitution in the zinc-binding site (H233Y, E230Q). The crystal of LHn/D diffracted at 2.3 Å resolution in space group *P*6_4_22 with cell dimensions a = b = 173, c = 222 Å; α = β = 90, γ = 120° (Table 1). Despite the large unit cell, a single molecule was present in the asymmetric unit corresponding to an unusually high solvent content of 75%. The X-ray crystal structure presents the two domains, LC and Hn, in a close interaction. No electron density was observed for regions 442–457 (between the two domains), residues 494–510, and the C-terminal poly-histidine tag (last fifteen residues), all in solvent-accessible areas (Fig. 1).

LHn/D share 34 to 35% identity with the corresponding fragment of BoNT/A, /B and, /E, for which the full length structures are available^19^,^20^,^21^. LHn/D presents a similar overall fold as previously observed for LHn/A and /B^17^,^18^. The translocation domain of serotype D is shown for the first time and includes two long coiled-coil helices with additional short helical segments on both sides. The catalytic domain has the globular fold typical of other light chains but shows key differences in flexible loop regions. Additionally, one of the most intriguing features of BoNT, the ‘belt’ region (residues 458–547), which is part of Hn and surrounds LC, was visible in the electron density map (despite high solvent content in the crystal) in the structure presented here and highlights the intricate interactions between the two domains. A PISA^22^ analysis shows that the LC-belt interaction is supported by over 40 potential hydrogen bonds and 2 salt bridges, making up a 3373 Å^2^ contact area. Additionally LC and Hn (excluding the belt) make a further 1392 Å^2^ buried interface area principally involving hydrophobic interactions.

### The light chain

**Figure 2:**
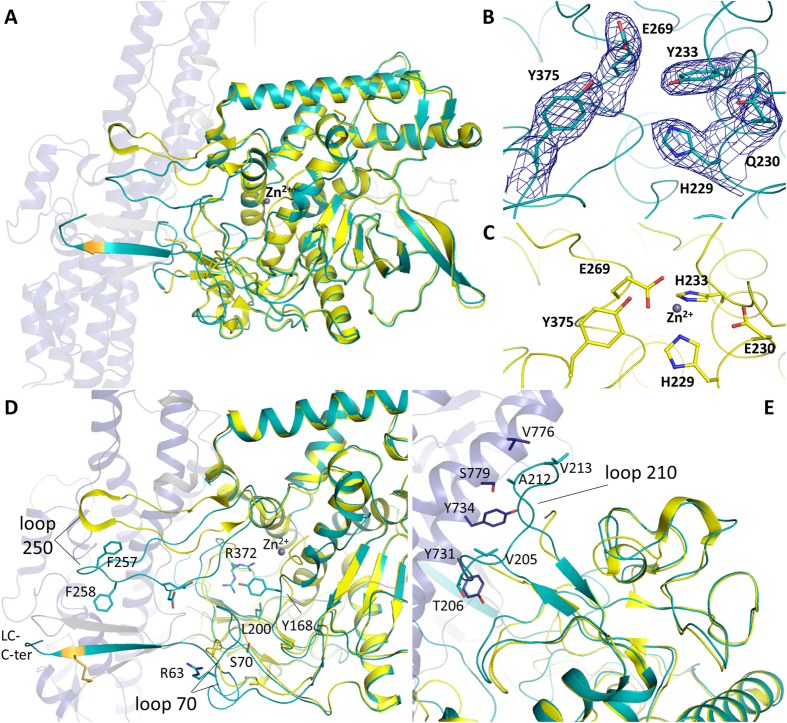
Comparison of the LC structure of LHn/D with LC/D. (**A**) Ribbon diagram representation of LHn/D structure with its light chain in cyan, belt region in grey and translocation domain in blue. PDB 2FPQ^23^ is shown in yellow with the zinc ion as a grey sphere. (**B**) Residues of the catalytic pocket of LHn/D are shown with sticks to highlight the effect of the double mutation E230Q and H233Y. The *2Fo-Fc* electron density map countered at 1σ level is shown in blue. (**C**) Residues of the catalytic pocket of LC/D presenting the classic zinc coordination motif HEXXH. (**D**) and (**E**) Close-up views of loop 250 and 210, respectively. Residues involved in loop interactions are shown in sticks and labelled.

The structure of the catalytically inactive mutant light chain superposes well with the high-resolution structure of a recombinant form previously reported (PDB code 2FPQ^23^) with a root mean square deviation (rmsd) of 2.3 Å (over 411 Cα atoms; Fig. 2). Loss of the zinc ion was confirmed with a local rearrangement of the active site residues (Fig. 2B,C). Mutation of the zinc-coordinating H233 to tyrosine caused displacement of the catalytic ion, replaced in its position with the Y233 hydroxyl group. Overall, the role of the zinc ion appears mostly catalytic with little impact on the structural integrity of the light chain.

The 2FPQ^23^ structure (hereafter referred to as ‘2FPQ’) corresponds to residues 1–424 of the light chain and thus do not include some of the elements interacting with the rest of the toxin. In this structure, residues 166–170 of the catalytic channel appear in two possible conformations, one is a random coil close to the zinc atom and the second a short β-strand completing the catalytic pocket. Furthermore, residues 173–179 were disordered. The present LHn/D structure, in contrast, was stable across residues 166 to 179 where segments 168–172 and 178–184 take on an α-helical arrangement (Fig. 2D). The multiple conformations of this area across the crystal structures, in vicinity of the active site, indicate a degree of flexibility that may provide the necessary space for substrate interaction^24^.

The most noticeable differences between the new structure and 2FPQ reside in flexible loop regions, particularly those at the interface with the translocation domain. For example, loop 63–70, which is surface accessible in 2FPQ, still presents a random coil in LHn/D but is stabilised by the extended carboxyl-terminal end of LC (residues 429–431) on one side (S70) and by the end of the ‘belt’ (F539) on the other (R63) (Fig. 2D). Additionally, loop 250, which presents low sequence identity with other light chains^23^ but is visible in 2FPQ, adopts a different conformation in LHn/D with a 10 Å and 25° movement (Fig. 2D). The loop is stabilised through hydrophobic interactions involving residues 256–258 (GFF) with Hn, and potential electrostatic interactions of residues Q260-D261 with the belt which occludes the catalytic pocket. A third area of interest is the flexible loop at positions 205–217 which was completely disordered in 2FPQ and is visible in LHn/D. The hydrophobic part of the loop (V205-T206) fits within the aromatic pocket composed of Y731 and Y734 of one of the long Hn helices, while the backbone of residues 212–214 is stabilised by hydrophobic interactions with the other helix (V776-S779) (Fig. 2E). Although crystal packing might influence some of these interactions, the observed changes could partly be attributed to the complex interface between the two domains compared to the structure of the single light chain (in 2FPQ).

The interaction between the catalytic domain and its VAMP substrate involves several binding sites based on the information provided by the structure of LC/F with a VAMP-inhibitor complex^25^ and recent mutagenesis work^24^. Several sub-sites of the catalytic pocket of LC/D have been shown to be essential for substrate binding. Interestingly the LHn/D structure highlights the plasticity of LC within the context of the whole toxin at these particular sites. For example R372 of S2′ (Fig. 2D) presents a double conformation but is expected to form a hydrogen bond with the P2′ site of VAMP. Additionally, the hydrophobic S1′ pocket and the S3 loop of the catalytic domain in LHn/D present new alternative conformations which were shown to cause at least a 50-fold loss of catalytic activity when mutated to Y168A/L200D and R63A, respectively (Fig. 2D)^24^.

**Figure 3:**
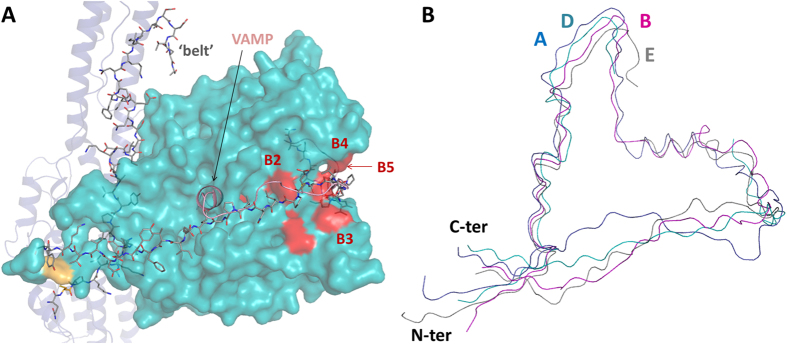
The belt of BoNT/D. (**A**) Crystal structure of LHn/D with the belt region represented with grey sticks. The rest of the translocation domain is shown in blue, the light chain in shown as a cyan surface. The structure of LC/F in complex with a VAMP peptide inhibitor (PDB 3FIE^25^) was superposed to LHn/D and VAMP is represented as a pink ribbon. (**B)** Superposition of the belt regions of LHn/A (PDB 2W2D^17^), /B (PDB 2XHL^18^), /D and BoNT/E (PDB 3FFZ^21^), in blue, magenta, cyan and grey ribbon, respectively. The amino- and carboxyl-end of the belt are labelled.

The long substrate requirement of all BoNT serotypes is atypical to other zinc proteases and involves several binding sites distal from the catalytic pocket^26^. Guo and Chen identified and labelled these exosites B1 to B5 in LC/D^24^. None of these sites present any conformational changes in LHn/D. While B1 is buried within LC, 15 Å away from the active site, the hydrophobic patches B4 to B5 show the extent of substrate recognition at the accessible surface of the free light chain. In LHn/D these pockets are interacting with the belt (Fig. 3) and implies a potential substrate inhibiting role for this region as also hypothesised for BoNT/A and /B^27^.

### The translocation domain and the belt

The catalytic domain is normally linked to the translocation domain through a single disulphide bridge after activation of BoNT into a di-chain molecule. Although the construct used for crystallisation experiment was not activated, residues 442–457, corresponding to the engineered cleavage site and flexible linker between the two domains, were not visible. The cysteine bridge is however stabilised by formation of a small β-sheet between the C-terminus of LC, the N-terminus of Hn and the end of the belt segment (Fig. 1).

The belt region in /D is overall similar to the corresponding segments in serotypes /A, /B and /E (Fig. 3). It surrounds LC following a hydrophobic groove along its surface. Interestingly, the belt in the other known BoNT structures has been observed in its entirety and takes on a conserved α-helical arrangement at a position where LC/A was shown to bind its substrate (SNAP-25) in a similar secondary conformation^21^,^26^. In LHn/D, seventeen amino acids of this area are missing altogether (residues 494–510), indicative of a flexible loop region which is unlikely to interact with LC/D. Remarkably, LC/D is known to recognise a shorter substrate than its counterparts^28^, and this site is therefore not expected to play a role in VAMP binding. The full function of the belt region in BoNT is not yet completely understood but evidence suggests a possible role beyond protection of LC, and towards a chaperone-like mechanism for translocation and membrane insertion^27^,^29^.

**Figure 4:**
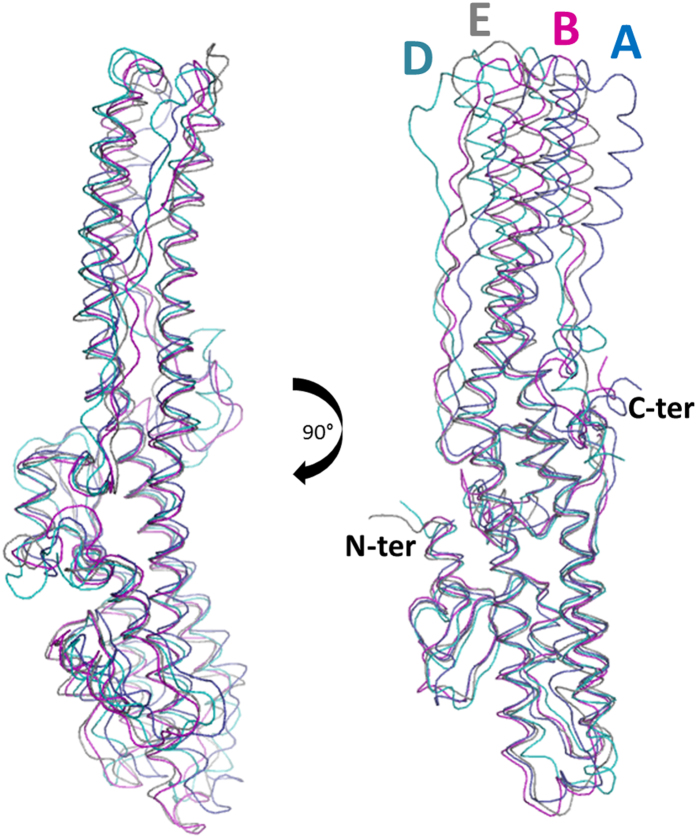
Comparison of BoNT translocation domains. Superposition of the translocation domains (without the belt) of LHn/A, /B, /D and BoNT/E, in blue, magenta, cyan and grey ribbon, respectively. The amino- and carboxyl-end of the domain are labelled.

**Figure 5:**
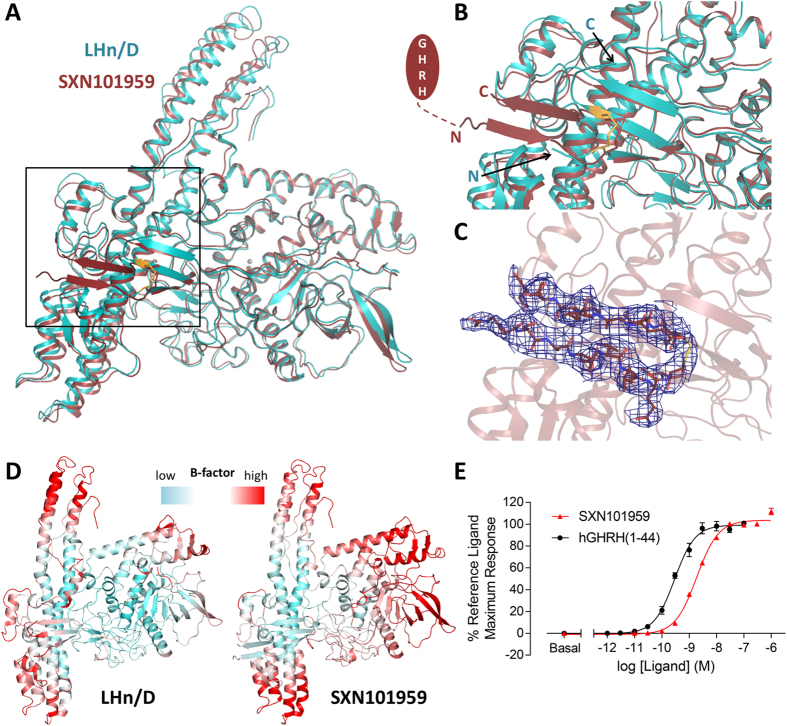
Crystal structure of SXN101959 and comparison with LHn/D. (**A**) The crystal structure of SXN101959, in dark red, was superposed with LHn/D, in cyan. The disulphide bridge between LC and Hn is highlighted in orange. The zinc ion of SXN101959 is shown as a grey sphere. (**B**) Close-up view of the domain interface in SXN101959 and LHn/D. The C-terminal end of LC and N-terminus of Hn are labelled. The location of the targeting ligand is represented with the missing part of the linker shown as a dashed line. (**C**) The visible part of the linker region of SXN101959 is highlighted with the *2Fo-Fc* electron density map countered at 1σ level shown in blue. (**D**) Temperature factor (B-factor) analysis. Ribbon diagram representation of LHn/D and SXN101959 structures, with a gradient colouring from low (cyan) to high (red) B-factors. Gradients were adjusted independently to highlight the less ordered area for each structure. (**E**) **Activation of the human GHRH receptor**. The receptor activation was detected by the intracellular accumulation of cAMP in CHO-K1-hGHRH-R cells incubated with hGHRH(1–44) (•) or SXN101959 (
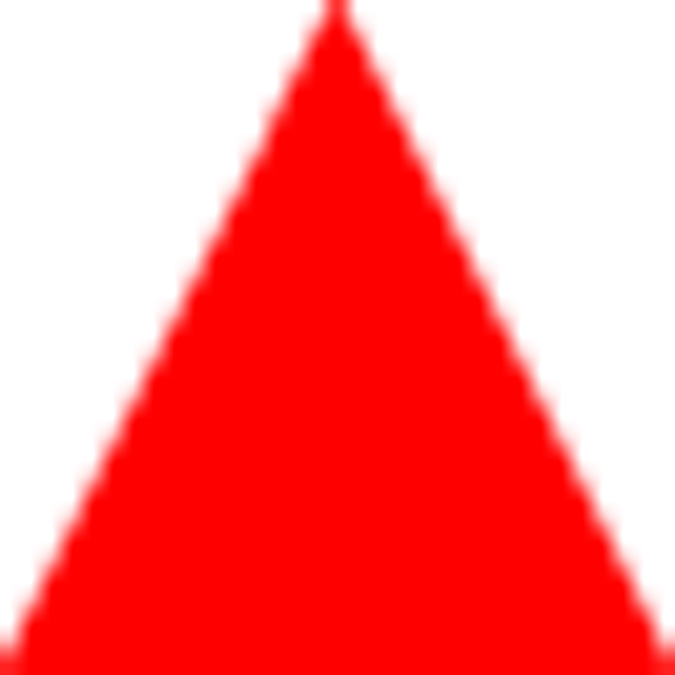
). Data are mean ± sem of triplicate samples from one experiment.

The main components of the translocation domain are the two coiled-coil helices of approximately 105 Å (Fig. 1). While the secondary α-helical structure is conserved across serotypes, the two helices all have a central bending point which curves to various degrees (Fig. 4). Of the four serotypes (with known Hn structures thus far), D appears the straightest. It should also be noticed that Hn/D presented particularly high B-factor at the extremities of these helices (Fig. 5D), with weak electron density for the random-coiled loop linking them. This is likely an indication of flexibility within these regions. The smaller helices flanking the main frame are also well conserved and similarly positioned across serotypes. Additionally the visible C-terminal end forms a small α-helix which is involved in crystal packing with a symmetry-related molecule and appears in a different orientation to the similar segment in LHn/A and /B.

### Structure of SXN101959

SXN101959 differs from LHn/D, and from natural BoNT molecules at the linker region between the two domains. It includes a Factor Xa cleavage site, a GHRH ligand domain (qGHRH[1–40]) and a GGGGS repeat (totalling an additional 56 residues). The engineered protein was crystallised as an activated di-chain molecule and diffracted up to 3.1 Å resolution (Table 1). The two molecules of the asymmetric unit are overall similar (rmsd of 1.05 Å over 828 Cα atoms) and present the same disordered regions which correspond to 66 amino acids of the linker peptide (described above), and 17 residues of the belt region. Both these areas were also not visible in LHn/D (Fig. 5).

Although the targeting ligand could not be observed, the crystal structure still provides valuable information on LHn/D as a framework for protein engineering. Indeed addition of a large peptide segment at the LC-Hn interface, which is essential for BoNT activity^3^, did not affect the LHn/D scaffold (each molecule superposes to LHn/D with rmsd of 1.08 (821 Cα) and 1.40 Å (828 Cα atoms), respectively). The light chains of SXN101959 and LHn/D are identical, with little variation in the solvent accessible loop regions. No noticeable difference was seen in the backbone of the active site (Fig. 5) despite SXN101959 being catalytically active. Clear electron density provided evidence of the zinc ion at the catalytic site, which is coordinated as per the classical HEXXH motif. Unsurprisingly, the more noticeable difference between SXN101959 and LHn/D resides in the translocation domain where the extremities of the coiled-coil helices are showing signs of flexibility, confirming what was observed in LHn/D (Fig. 5).

The linker region (residues 438–513 in SXN101959) was engineered to provide ease of access of the targeting GHRH ligand for its receptor. It is flanked with an N-terminal protease cleavage site that allows activation of the whole molecule into its di-chain form, and frees the amino end of the peptide chain. The biological activity of GHRH is retained by the 29 N-terminal amino acids for recognition by the GHRH receptor^30^. In order to give the system more structural flexibility, three GGGGS repeat motifs were included on the C-terminal side of the peptide. This allows for the targeting peptide to be optimally and centrally presented while it remains covalently linked to the translocation domain. This flexible segment, was disordered in the crystal structure. It is located in a solvent accessible area where it could be easily accommodated within the crystal packing (Supplementary Figure). It should also be noticed that the observable part of the linker, consisting of the LC’s carboxyl and Hn’s amino ends (438–443 and 509–513, respectively), is stabilised through the formation of an anti-parallel β-sheet conformation (Fig. 5) so that one of the GGGGS repeats is visible. The targeting peptide is not interacting with the rest of the molecule, which is encouraging from a biological perspective, as it relies on this freedom of movement to activate the GHRH receptor. SXN101959 is known to activate GHRH receptor in rat primary pituicytes which leads to internalisation and intracellular cleavage of VAMP3^14^.

Integrity of the sample used for structural study was tested by measuring the intracellular accumulation of the second messenger cAMP in human GHRH receptor-expressing cells. SXN101959 elicited a concentration-dependent increase in cAMP accumulation with a pEC50 (potency) of 8.73, compared with the pEC50 of hGHRH(1–44) of 9.54. The maximum response of SXN101959 was the same as hGHRH(1–44), indicating that the TSI is a full agonist of hGHRH receptor. Activation of the target receptor confirms the presence of the qGHRH(1–40) ligand within the structure of SXN101959 (Fig. 5E). Thus the crystal structure of SXN101959 highlights the validity of LHn/D in the context of a biologically active TSI.

### Small-angle X-ray scattering studies on LHn

**Figure 6:**
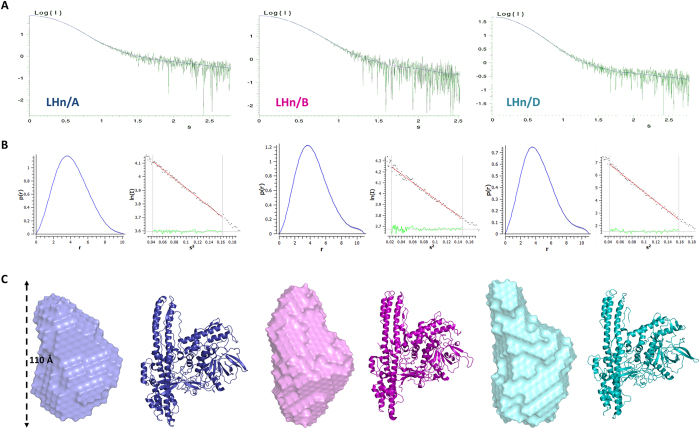
Small-angle X-ray scattering (SAXS) on LHn. (**A**) Scattering curve of LHn/A, /B and /D, from left to right. (*I* is a relative unit, *s* is expressed in nm^−1^). (**B**) The interatomic distance function (*P(r)*) was calculated (x axis, r in nm) with GNOM^43^ (left) and Guinier plot is presented for each samples. (**C**) *Ab initio* solution scattering models. SAXS bead models were generated with DAMMIF^44^ and averaged in DAMAVER^45^. The models are compared to the samples’ respective X-ray crystal structures.

The botulinum neurotoxin has been extensively studied by X-ray crystallography but little information is available on the relevance of these structures in solution. Small-angle X-ray scattering (SAXS) experiments were carried out on LHn/A, /B and /D. All data were collected on station X33 at DESY (Germany) and the solution scattering curves are provided in Fig. 6A. The purified samples were all in their activated di-chain form.

A Guinier analysis of the scattering curves was consistent with the distance distribution function (*P(r)*) in determining the radius of gyration (*R*_*g*_) for each LHn serotypes with values of approximately 32, 34 and 34 Å for A, B and D, respectively (Table 2; Fig. 6B). Interestingly, LHn/B and /D also share a similar maximum dimension (*D*_*max*_) of 110 Å, slightly longer than LHn/A at 102 Å. The comparable features of the three samples correlate with the known crystal structures. The theoretical scattering curves extrapolated from the high-resolution structures were calculated with CRYSOL^31^ and fitted well with the experimental values (Table 2) although the corresponding *R*_*g*_ were somewhat lower than the experimental ones (between 31 and 32 Å). *Ab initio* models were generated from the scattering curves (Fig. 6C, Table 2), and present the overall shape of LHn in solution.

Manual docking allowed the comparison between the *ab initio* bead models and the crystal structures. The distinctive shape of the two domains forming the LHn molecules can be clearly noticed in all three samples. Indeed the atypical long coiled-coil helices of the translocation domain give the models an extended form over 100 Å long whereas the light chain accounts for the globular central shape. In accordance with the statistical comparison (Table 2), LHn/B appears to show the best resemblance between crystal and solution structures. The smaller dimensions of LHn/A in solution might be the reflection of the more pronounced central kink of its translocation domain (Fig. 4). Additionally, LHn/D presents a less defined upper region which might be an indication of domain movement, considering the bead model represents an averaged form of the solution state. Interestingly the extremities of the translocation domain were also showing signs of mobility in the crystal structures of LHn/D and SXN101959.

Overall, the information collected for LHn/A, /B and /D showed that the fragments have conserved the LHn fold in the solution state although the resolution of the data may not allow the visualisation of small variations.

## Conclusion

Botulinum neurotoxins (BoNTs) are some of the most potent protein toxins and their modular architecture have made them molecules of choice for the design of a novel class of biopharmaceuticals labelled Targeted Secretion Inhibitors. The intracellular activity of BoNT towards members of the soluble N-ethylmaleimide-sensitive factor-attachment protein receptor family, a critical element of vesicular secretion, can be redirected towards specific cell types. This concept relies on the functionality of the LHn fragment which consists of the catalytic and translocation domains of BoNT. The distinct but variable properties of each serotype, such as substrate specificity and duration of action, have led to the development of tailor-made TSI with optimised applicability. Recent report of the biological activity of SXN101959 in inhibiting GH secretion has demonstrated the significance of using LHn/D as a framework for protein engineering. The crystal structures of the LHn fragment and SXN101959 present for the first time the properties of the combined translocation and catalytic domains of BoNT/D. The data provided have highlighted the plasticity of the light chain and its extensive interaction with the translocation domain. The translocation domain appears as a ‘solid scaffold’ on which to build on a retargeting partner. Indeed the structure of SXN101959 showed that addition of a long peptide ligand in a central position did not cause any significant structural conformational changes, thus demonstrating the robustness of LHn structural framework, and its ability to accommodate novel arrangements of the domain subunits, such as central presentation, within TSI proteins. Finally, the solution scattering analysis confirmed the structural integrity of the LHn fragment across serotypes. Altogether this study has provided useful information on the structure of BoNT/D, and the LHn fragment in general, that will be essential for the development of the next generation of protein therapeutics based on the TSI technology.

## Methods

### LHn/A, /B and /D cloning, expression and purification

Cloning, expression and purification were carried out following methods described previously^17^,^18^. Briefly, LHn constructs were cloned into modified pET26b expression vector (Novagen, Madison, WI, USA) with a C-terminal 6 x His-tag and expressed into *E. coli* BL21 (DE3) cells. The LHn genes were engineered to encode for an enterokinase or factor Xa cleavage site between the LC and Hn domains. Proteins were purified by affinity chromatography using Ni^2+^-charged chelating sepharose column (GE Healthcare, UK) and hydrophobic interaction (Phenyl sepharose column, GE Healthcare, UK). Samples were kept in 0.05M HEPES pH 7.2 with 0.2 M NaCl.

The LHn/D construct used for X-ray crystallography correspond to the catalytically inactive LHn/D double-mutant E230Q and H233Y. Standard site directed mutagenesis techniques employing PCR amplification and subsequent sequencing were used to create the mutated DNA construct. In addition to the mutations required to eliminate catalytic activity, this construct contains an engineered enterokinase cleavage site between LC and Hn but was kept as a single chain sample for crystallisation. Expression and purification were performed as per the other LHn samples described above.

### SXN101959 preparation

This protein was prepared as previously reported^15^. SXN101959 was designed using the LC and Hn of BoNT/D (Entrez protein database accession number P19321), a GHRH ligand domain, a lead sequence spacer, and a factor Xa protease activation site located between the two domains, as previously reported.

### GHRH receptor activation assay (cAMP second messenger accumulation)

Frozen CHO-K1 cells recombinantly expressing the human GHRH receptor were rapidly thawed, collected by centrifugation and re-suspended in assay buffer Hams F-12 (Life Technologies) , 5 mM HEPES (Life Technologies), 1 mM 3-isobutyl-1-methylxanthine (IBMX, from Calbiochem), 0.05% BSA (Perkin Elmer), pH 7.4). Cell suspension (5,000 cells well-1) was incubated in white 384-well microplates in triplicate with increasing concentrations of human GHRH(1–44, from Bachem) (final concentration 1 pM—100 nM) or SXN101959 (10 pM—1 μM), which had been serially diluted in buffer. Plates were incubated at 21 ± 3 °C for 90 minutes. Following ligand incubation, detection mix (consisting of cell lysis buffer, Lance Eu-W8044–labelled streptavidin, cAMP-biotin, and Alexa Fluor 647–anti-cAMP antibody) (kit from Perkin Elmer) was added to each well. Plates were incubated at room temperature for 24 hours, and the fluorescence emitted at 665 and 615 nm following excitation at 320 nm was measured using an EnVision microplate reader (PerkinElmer). Raw data was plotted as 615/665 nm emission ratio and converted to percentage of the maximum response (Emax) defined by hGHRH(1–44). Dose response curves were fitted using a variable slope model (using GraphPad Prism software) using equation (1).
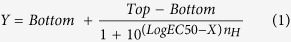
where Y is the RFU, top and bottom are plateaus in the units of the Y axis, X is the log molar concentration and n_H_ is the Hill slope. EC50 value is the negative log of the mid-point location of the curve.

### X-ray crystallography

Crystals of LHn/D were grown with 2 μl of protein at 56 mg/ml mixed with 1 μl of reservoir solution consisting of 30% v/v pentaerythritol ethoxylate (15/4 EO/OH), 0.1 M HEPES 7.5, 6% w/v polyvinylpyrrolidone (MIDAS D2, Molecular Dimensions) and suspended above the well as a hanging drop. Crystals grew within 7–10 days into pear-like shapes of 50 to 500 μm in length.

SXN101959 crystals were grown from the sitting drop method against 0.1 M sodium actetate pH 5.5, 0.8 M sodium formate, 10% w/v polyethylene glycol 8000, 10% w/v polyethylene glycol 1000 (Clear Strategy Screen 1, B6, Molecular Dimensions). A drop of 200 nl of protein at 10 mg/ml was mixed with equal amount of reservoir and incubated at 16 °C.

Diffraction data were collected at station I03 of the Diamond Light Source (Oxon, UK), equipped with a PILATUS-6M detector (Dectris, Switzerland). A complete dataset to 2.3 Å and 3.1 Å were collected from single crystals at 100 K for LHn/D and SXN101959, respectively. Raw data images were processed and scaled with DIALS^32^, and AIMLESS^33^ using the CCP4 suite 6.5^34^. The resolution cut-off chosen was based on the CC_1/2_^35^ as adding weak high-resolution data beyond the commonly used limits recorded on the highly sensitive detector showed significant improvements in electron density maps. For LHn/D, initial molecular replacement was performed with the coordinates of LC/D (PDB code 2FPW^23^) to determine initial phases for structure solution in PHASER^36^. The translocation domain was built in from a partial poly-alanine model of the secondary structures of LHn/B (PDB code 2XHL^18^) and extended with BUCCANEER^37^. The refined LHn/D structure was then used for molecular replacement to solve SXN101959. The working models were refined using REFMAC5^38^ and manually adjusted with COOT^39^. Water molecules were added at positions where Fo−Fc electron density peaks exceeded 3σ, and potential hydrogen bonds could be made. Validation was performed with MOLPROBITY^40^. Crystallographic data statistics are summarized in Table 1. All figures were drawn with PyMOL (Schrödinger, LLC, New York).

### Small-angle X-ray scattering

Protein samples of LHn/A, LHn/B, and LHn/D were kept in 0.05 M HEPES 7.2, 0.2 M NaCl for SAXS analysis and checked for monodispersity by dynamic light scattering (Malvern Instruments). Samples were diluted to give three final concentrations at 5, 2.5 and 1 mg/ml. These dilutions were checked by absorbance measurement at 280 nm using a NanoDrop ND-1000 (Thermo Scientific), and concentration discrepancies were corrected for data analysis.

Solution scattering (SAXS) data for the three LHn molecules were collected at DESY, (Germany), EMBL Hamburg, beamline X33^41^. For each sample, a buffer measurement was performed before and after the sample at room temperature, using a PILATUS-1M (Dectris, Switzerland) detector. SAXS measurements were performed for each sample at three concentrations. Calibration was carried out with BSA at 5 mg/ml in 0.05 M HEPES pH 7.5 as a molecular weight standard. The data were processed and averaged with PRIMUS^42^ followed by particle distance distribution function *P(r)* analysis using GNOM^43^. Data were further treated by using DAMMIF^44^ to produce twenty *ab initio* models that were averaged with DAMAVER^45^.

## Additional Information

**How to cite this article**: Masuyer, G. *et al*. Structural analysis of *Clostridium botulinum* neurotoxin type D as a platform for the development of targeted secretion inhibitors. *Sci. Rep*. **5**, 13397; doi: 10.1038/srep13397 (2015).

## Supplementary Material

Supplementary Information

## Figures and Tables

**Table 1:** X-ray crystallography: data collection and refinement statistics.

	LHn/D	SXN101959
Resolution (Å)^a^	2.3 (2.3–2.34)	3.1 (3.1–3.25)
Space group	*P6*_*4*_*22*	*P2*_*1*_*2*_*1*_*2*_*1*_
Cell dimensions (Å; a, b, c); angle (°; α, β, γ)	173, 173, 222; 90, 90, 120	88, 144, 173; 90, 90, 90
Total/Unique reflections	321,682/86,306	122,006/35,774
Completeness (%)^a^	98.8 (97.7)	89.0 (81.2)
*R*_merge_^a^,^b^	0.059 (1.09)	0.127 (1.02)
*R*_pim_^a^,^c^	0.048 (1.04)	0.101 (0.859)
I/σ(I)^a^	8.1 (0.5)	6.0 (1.0)
CC_1/2_^d^	0.998 (0.214)	0.990 (0.331)
Multiplicity	3.7 (2.6)	3.4 (2.6)
*R*_*cryst*_^e^	22.0%	25.0%
*R*_free_^f^	24.7%	29.5%
Rmsd in bond lengths (Å)	0.011	0.007
Rmsd in bond angles (°)	1.45	0.97
B- factor statistics (Å^2^)		
Protein all atoms	79.1	94.6/110.3^g^
Protein main chain atoms	78.3	95.0/110.7
Protein side chain atoms	79.9	94.2/109.8
Zinc ion	N/A	64.9/85.4
Solvent atoms	66.4	51.0
Ramachandran statistics (Molprobity)		
Favoured	96.2%	93.9%
Outliers	0.2%	0.5%
PDB code	**5BQN**	**5BQM**

^a^Values in parentheses refer to the highest resolution shell.

^b^*R*_merge _= ΣΣ_*i*_|*I*_*h *_− *I*_*hi*_|/ΣΣ_*i*_*I*_*h*_, where *I*_*h*_ is the mean intensity for reflection *h*.

^c^*R*_pim _= Σ_*h*_(1/*n*_*h *_− 1) Σ_*l*_|*I*_*hl *_− (*I*_*h*_)|/Σ_*h*_Σ_*l*_(*I*_*h*_).

^d^Correlation coefficient between random half datasets^35^.

^e^*R*_cryst _= Σ‖*F*_*o*_|_* *_− |*F*_*c*_‖/Σ|*F*_*o*_|, where *F*_*o*_ and *F*_*c*_ are measured and calculated structure factors, respectively.

^f^*R*_free _= Σ‖*F*_*o*_|_* *_− |*F*_*c*_|/Σ|*F*_*o*_|, calculated from 5% of the reflections selected randomly and omitted during refinement.

^g^The two B-factor values recorded here correspond to two molecules in the asymmetric unit.

**Table 2:** Small angle X-ray scattering data statistics for LHn serotypes /A, /B and /D.

	Guinier^1^	*P(r)*function^1^		Crystal structures^2^		Structural modeling^3^	
	*R*_*g*_ (Å)	*R*_*g*_ (Å)	*D*_*max*_(Å)	*R*_*g*_ (Å)	Fit*(χ*^2^)	Discrepancy*(χ*^2^)	NSD
**LHn/A**	32.1	32.0	102	31.3	1.5	0.88	0.60
**LHn/B**	33.9	33.9	110	32.3	1.0	0.81	0.60
**LHn/D**	33.7	33.7	110	31.4	2.0	0.97	0.62

^1^The Guinier analysis and distance distribution function (*P(r)*) were performed with PRIMUS^42^ and used to calculate the maximum dimension (*D*_*max*_) and the radius of gyration (*R*_*g*_).

^2^The X-ray crystal structures of LHn/A, /B and /D were used for theoretical scattering calculation and compared to experimental data using CRYSOL^43^.

^3^The discrepancy (*χ*2) between experimental and *ab initio* data was calculated by the program DAMMIF^44^ with the average of 10 models (NSD = normalized spatial discrepancy).
