# Supplementary material for: Structural analysis of Clostridium botulinum neurotoxin type D as a platform for the development of targeted secretion inhibitors
Source: Sci Rep. 2015 Sep 1;5:13397. doi: 10.1038/srep13397 (PMC4555039; doi:10.1038/srep13397)

Supplementary Information

Structural analysis of *Clostridium botulinum* neurotoxin type D as a platform for the development of targeted secretion inhibitors

Geoffrey Masuyer[1,3], Jonathan R. Davies[1], Kevin Moore[2]

John A. Chaddock[2] and K. Ravi Acharya[1]

[1] Department of Biology and Biochemistry, University of Bath, Claverton Down, Bath BA2 7AY, UK

[2] Ipsen Bioinnovation Limited, Units 4-10, The Quadrant, Barton Lane, Abingdon, Oxon OX14 3YS, UK

[3]Current address: Department of Biochemistry and Biophysics, Arrhenius Laboratories for Natural Sciences, Stockholm University, 10691 Stockholm, Sweden

Correspondence and requests for materials should be addressed to K.R.A. (email: bsskra@bath.ac.uk)

Supplementary Figure

Crystal packing of LHn/D and SXN101959 within their unit cell. (**A**) Crystal structure of LHn/D is in space group $P6_422$ with cell dimensions a=b=173, c=222 Å; α=β=90, γ=120° with a single molecule per asymmetric unit and a solvent content of 75 %. Unit cell is drawn in cyan. (**B**) Crystal structure of SXN101959 is in space group $P2_12_12_1$ with cell dimensions a=88, b=144, c=173Å; α=β=γ=90° and two molecules per asymmetric unit with 54% solvent content. Unit cell is drawn in grey. (**C**) Close up of the crystal packing showing the free space where the qGHRH ligand domain should be located. Positions of the ligand for each molecule of the asymmetric unit are shown with an asterisk. The cysteine bridge between the two domains is highlighted in yellow

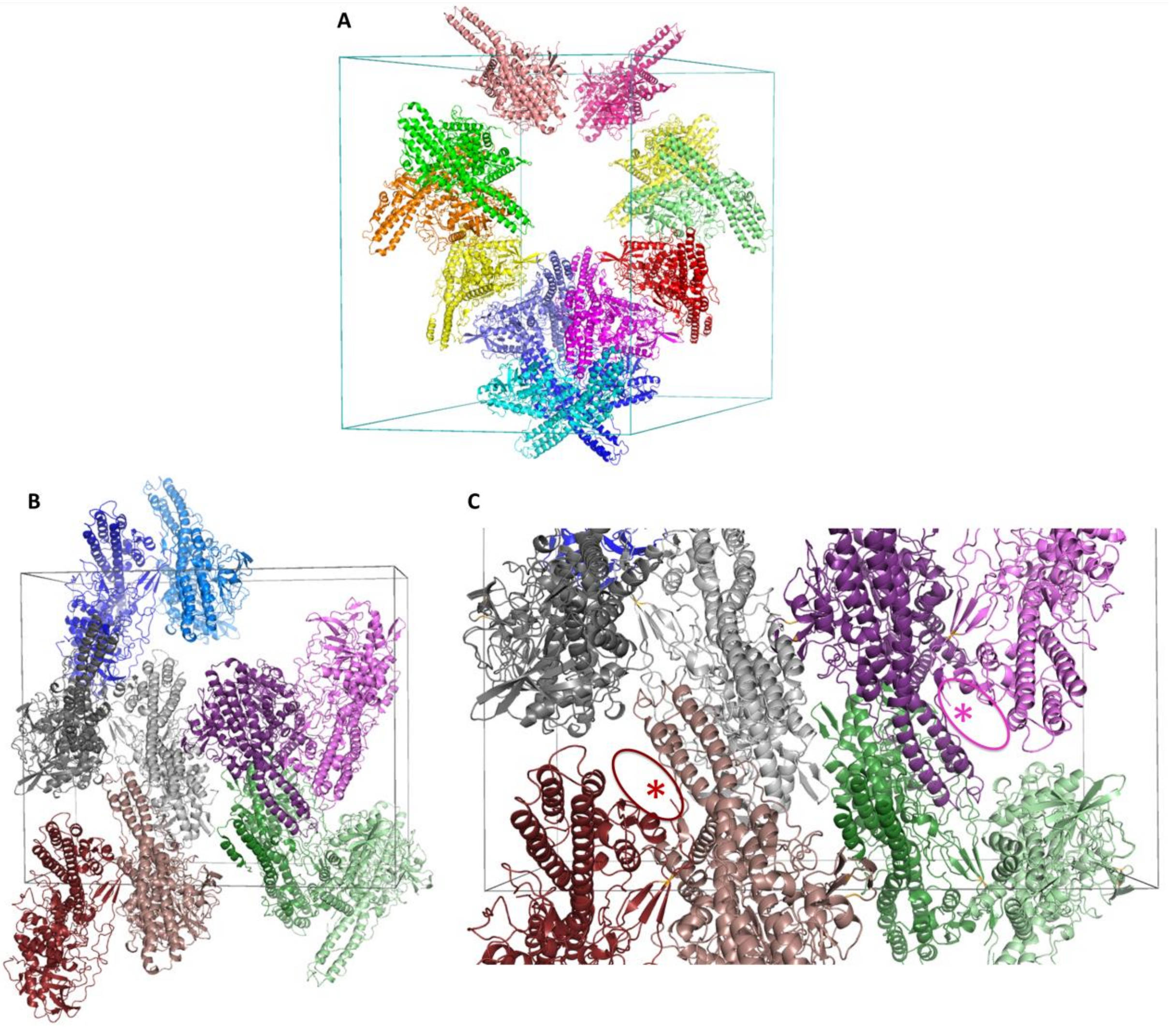

Supplement: Supplementary Information [file srep13397-s1.pdf]
